# Unravelling the Genotype of the Apical Variant of Hypertrophic Cardiomyopathy in a Swedish Cohort

**DOI:** 10.3390/genes16050494

**Published:** 2025-04-26

**Authors:** Antheia Kissopoulou, Rada Ellegård, Eva Ingemarsdotter Fernlund, Jan-Erik Karlsson, Henrik Green, Cecilia Gunnarsson

**Affiliations:** 1 Department of Internal Medicine, County Council of Jönköping, Department of Health, Medicine and Caring Sciences, Linköping University, 58185 Linköping, Sweden; jan-erik.karlsson@rjl.se; 2 Department of Clinical Genetics, and Department of Biomedical and Clinical Sciences, Linköping University, 58185 Linköping, Sweden; rada.ellegard@regionostergotland.se (R.E.); cecilia.gunnarsson@regionostergotland.se (C.G.); 3 Department of Biomedical and Clinical Sciences, Division of Pediatrics, Linköping University, Crown Princess Victoria Children’s Hospital, Linköping University Hospital, 58185 Linköping, Sweden; eva.fernlund@regionostergotland.se; 4 Department of Clinical Sciences Lund, Lund University, Skane University Hospital, Pediatric Heart Center, 22185 Lund, Sweden; 5 Division of Clinical Chemistry and Pharmacology, Department of Biomedical and Clinical Sciences, Linköping University, 58185 Linköping, Sweden; henrik.green@liu.se; 6 Department of Forensic Genetics and Forensic Toxicology, National Board of Forensic Medicine, 58185 Linköping, Sweden; 7 Centre for Rare Diseases in South East Region of Sweden, Linköping University, 58185 Linköping, Sweden

**Keywords:** apical hypertrophy, genetic variant, cardiomyopathy, sarcomere, apical aneurysm

## Abstract

Background: Apical hypertrophic cardiomyopathy (ApHCM) is a distinct variant of hypertrophic cardiomyopathy (HCM). Few studies have focused on the genetic determinants of this subtype. We aimed to investigate the genetic basis of apical hypertrophy in a Swedish cohort. Methods–Results: Longitudinal data on 58 unrelated index patients with ApHCM from the Southeast healthcare region in Sweden from 2010 to 2024 were assessed retrospectively. Additionally, the original raw data from genetic testing were re-evaluated using AI-based Emedgene software. Patients were 47 ± 14 years old, and 60% males. A total of 72.4% had the pure apical type and the remaining had the mixed phenotype, dominant distal. In the cohort, 50/58 (86.2%) underwent genetic testing, of whom 7/50 (14%) were considered genotype positive for a pathogenic/likely pathogenic variant, mainly in MYH7 (43%) and in the non-sarcomeric ALPK3 gene (28.6%). A re-evaluation of the original data from genetic testing identified a previously unreported variant in the skeletal muscle α-actin (ACTA1) gene. Overall, 21 of 58 patients (36.2%) had HCM-related events during their disease course: 10% had a stroke, and 12% had heart failure. Atrial fibrillation was present in 41.4% and non-sustained ventricular tachycardia occurred in 29.3% of the patients. Apical aneurysm was observed in 17.2% of cases. Patients with a positive genotype were more likely to have a positive family history of HCM compared to those with a negative genotype (*p* = 0.020). Conclusions: In ApHCM, a positive genotype was found less frequently compared to classic HCM. Only 14% of patients with ApHCM were found to be genotype positive, indicating that apical hypertrophy represents a genetically unique population with low risk of mortality. Nevertheless, patients with ApHCM faced higher rates of atrial fibrillation, ventricular arrhythmias, and apical aneurysms.

## 1. Background

**Figure 1:**
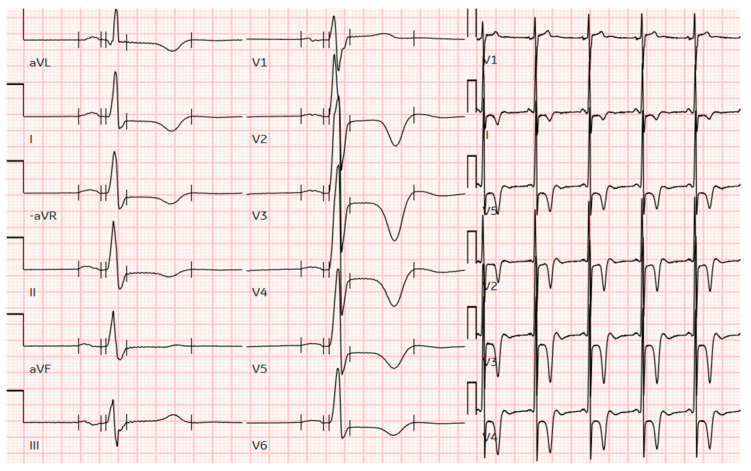
The ECG of a male patient from this cohort with pure apical hypertrophy of 16 mm demonstrates profound negative T-wave inversion in precordial leads and voltage criteria for LV hypertrophy. ECG, electrocardiogram; LV, left ventricular.

Apical hypertrophic cardiomyopathy (ApHCM) is a relatively rare morphologic variant of hypertrophic cardiomyopathy (HCM), characterized by unexplained left ventricular hypertrophy (LVH) predominating in the apex. The typical features of ApHCM, first described by Sakamoto in Japan in 1976 [1] and by Yamaguchi in 1979, also known as Yamaguchi syndrome [2], include “giant” T-wave negativity on the electrocardiogram (Figure 1) and a “spade-like” configuration of the left ventricular (LV) cavity at end-diastole observed on echocardiography. ApHCM accounts for up to 25% of HCM cases in Asian populations and 1% to 10% in non-Asian populations, with estimates of ApHCM ranging from 3% to 10% in Western patients [3].

As this subtype of HCM primarily affects the apex of the heart, it poses diagnostic challenges that can lead to delayed detection. Unlike other morphologic variants of HCM, ApHCM does not cause left ventricular outlet obstruction (LVOTO) but rather exists either with or without a mid-cavity obstruction or apical aneurysm. Therefore, patients often present with nonspecific symptoms, further contributing to later diagnosis. The appropriate management of ApHCM is crucial [3,4], given that it is associated with an increased risk of atrial fibrillation, ventricular arrhythmias, and additional risk for apical myocardial infarction, apical aneurysm development, and sudden cardiac death (SCD). However, risk stratification for these complications is more challenging due to the phenotypic variation in ApHCM. Despite these potential complications, ApHCM is generally characterized by a benign clinical course with favorable long-term prognosis [5,6,7].

There is no acceptable maximal wall thickness (MWT) threshold for diagnosing apical hypertrophic cardiomyopathy, with guidelines referring to ≥15 mm MWT for all hypertrophic cardiomyopathy subtypes [8,9,10,11]. A normal myocardium naturally tapers apically; a fixed diagnostic threshold fails to account for this. Using cardiac magnetic resonance (CMR), “relative” ApHCM has been described with typical electrocardiographic features, a loss of apical tapering, and cavity obliteration, but also with MWT < 15 mm. Relative ApHCM was defined as those with characteristic ApHCM imaging and electrocardiographic features but with a wall thickness < 15 mm. Relative ApHCM is believed to be an early ApHCM phenotype [3]. Hughes et al. [12] have highlighted that per-segment indexed apical wall-thickness thresholds are highly accurate for detecting apical hypertrophy, providing confidence to the reader to diagnose ApHCM in those not reaching current internationally recognized criteria. Early follow-up studies suggest progression is common, with 23% develop ApHCM overt within two years [13].

**Figure 2:**
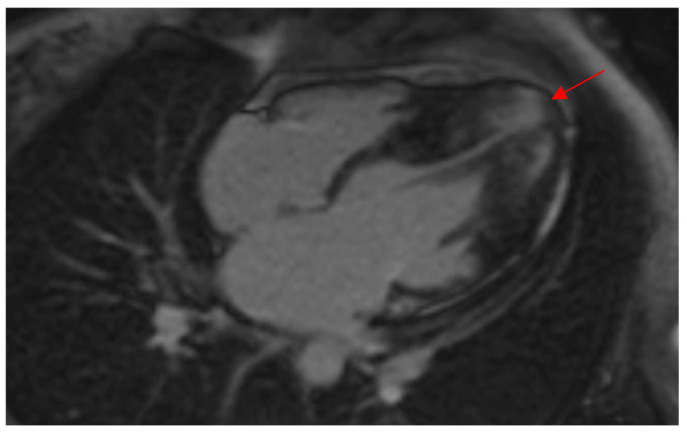
CMR demonstrates a thickened LV apex in a female patient of this study with wall thickness extending to the midventricular septum, indicating a “mixed ApHCM” (distal-dominant form). Note the presence of LGE in the hypertrophied apex (red arrow) as well as the apical aneurysm. ApHCM, apical hypertrophic cardiomyopathy; CMR, cardiac magnetic resonance imaging; LGE, late gadolinium enhancement; LV, left ventricular.

Morphologically, ApHCM can be classified into two groups: “pure” ApHCM, characterized by isolated asymmetric apical hypertrophy, and “mixed” ApHCM (distal-dominant form), where there is coexistent hypertrophy of the interventricular septum. Patients with the “mixed” ApHCM pattern have to demonstrate the greatest wall thickness in the apical segments (Figure 2). Recent studies [14,15] have shown that the occurrence of HCM-related adverse events was significantly higher in the distal-dominant group compared to the pure apical group.

As with classic HCM, identified pathogenic genetic variants in ApHCM are mainly sarcomeric, autosomal dominant, and influenced by environmental and ethnic/demographic factors including sex. ApHCM is characterized by occurrence at a later age, with a lack of genotype–phenotype correlation [16,17]. In ApHCM pathogenic sarcomere gene variants have been found to be present in up to 30% of the patient population [3,16,18]. A few select sarcomere protein gene defects (i.e., ACTC; p.Glu101Lys) have been reported in the apical HCM phenotype [16,19,20]. To date, no consistent genotype–phenotype correlation has been established, and most of the reports underscore the heterogeneity of the clinical disease expression in patients with pathogenic variants within the same sarcomere protein gene. The literature reporting sarcomeric pathogenic gene variants in patients with ApHCM is limited to a relatively small number of unrelated patients [16,17,18]. Moreover, patients with ApHCM are less likely to report a positive family history, suggesting differences in pathophysiology. Limited research has focused on the genetic factors underlying this subtype. Studies of asymmetric cardiomyopathy have been limited by cohort size and incomplete characterization by echocardiography, such that the genetics of patterns of increased mass are not thoroughly explored. Specific data regarding genetic profiling in the different ApHCM morphologies or ethnicities are lacking. In a study looking at genotype–phenotype correlations in ApHCM [17], those that carried a pathogenic sarcomere gene variant had a stronger family history of HCM, but no phenotypic features were significantly different. This study aimed to investigate the genetic basis and clinical characteristics of apical hypertrophy in a Swedish cohort. Understanding these patterns of regional hypertrophy may paint a fuller picture of cardiovascular disease outcomes and risk.

## 2. Methods

The longitudinal data of 58 unrelated index patients with an established diagnosis of ApHCM at the outpatient cardiogenetic clinic of the county Hospital Ryhov in Jönköping, Sweden and at the University hospital Linköping, Sweden from 2010 to 2024 were assessed retrospectively. Clinical, imaging, and functional parameters were examined. This was accomplished through a systematic and thorough review of medical history and medical records, both at the initial visit and at subsequent visits.

The patients with ApHCM had to meet the current diagnostic criteria for the ApHCM [9,10,11], which included the demonstration of asymmetric left ventricular hypertrophy (LVH), confined predominantly to the LV apex with an apical wall thickness ≥ 15 mm or ≥13 mm for familial hypertrophic cardiomyopathy (HCM) with and without electrocardiogram abnormalities suggestive of ApHCM. Secondary causes of left ventricular hypertrophy, such as arterial hypertension, structural heart disease, drug toxicity, and other multisystem diseases, such as metabolic, endocrinologic, neuromuscular, neurologic, genetic syndromes or phenocopies of HCM were excluded.

Information obtained from the medical records included demographics, clinical parameters, family history for HCM or SCD, genetic testing, and pharmacological and non-pharmacological treatments. Regarding the echocardiographic measurements, ApHCM was defined using the conventional criteria of MWT ≥ 15 mm in end-diastole measured manually and other characteristic features of the disease (distinctive electrocardiographic changes, apical cavity obliteration, or apical aneurysm). Left ventricular ejection fraction (LVEF), left atrial size (LA), and mitral regurgitation (MR) were measured as previously described. Most of the CMRs were performed using 3.0 Tesla CMR scanner. LV measures of geometry and function were analyzed using standardized protocols. Myocardial T1 mapping was used to assess for diffuse myocardial fibrosis, and late gadolinium enhancement (LGE) to assess myocardial fibrosis content.

Genetic testing using the approved clinical genetic panel for cardiomyopathy at the time of recruitment was performed for the index case. All patients included in this study provided informed consent for genetic testing and evaluation. Additionally, in 2025, raw data from all patients who had undergone genetic testing were re-processed using Sentieon followed by analysis using Emedgene (Illumina, San Diego, CA, USA). Candidate variants associated with the Human Phenotype Ontology—HPO cardiomyopathy (HP:0001638) were identified using Emedgene’s guided AI (XAI). Variants were assessed according to the guidelines set by the American College of Medical Genetics and Genomics (ACMG) [21]. The AI software used, Emedgene, is a commercially available AI model trained on known pathogenic variants that can be used to identify and rank germline variants with potential association with disease [22]. The software reduces the number of variants that need manual interpretation per patient as well as facilitating the ranking of candidate variants linked to a patient phenotype based on HPO terms, meaning that candidate variants outside of set gene lists can be identified. The implementation of Emedgene and similar AI tools to streamline variant interpretation in clinical settings is becoming increasingly more common. Patients with a positive genotype carried either pathogenic or likely pathogenic variants.

Clinical, genetic, and outcomes data on 58 patients with HCM are presented as frequency (and percentage) for non-continuous variables and mean ± SD for continuous variables where appropriate. Student’s t test was used for the comparison of continuous variables. Categorical variables were expressed as frequencies and percentages and were compared using Chi-square test or Fisher’s exact test for small samples, respectively. A *p*-value < 0.05 was considered statistically significant. Data analysis was performed using SPSS version 27. Complications and adverse event rates included heart failure, stroke, appropriate ICD shock, myocardial infarction, embolism, death, SCD, sustained VT, or resuscitated cardiac arrest.

The study protocol was approved by the Swedish Ethical Review Authority Dnr 2024-07927-02 and all individuals gave informed consent prior to enrolment. The study complies with the ethical guidelines of the 1975 Declaration of Helsinki.

## 3. Results

### 3.1. Clinical Findings of the Apical HCM Cohort

Baseline clinical characteristics, echocardiographic features at initial evaluation, and reasons for diagnosis are listed in Table 1 and Table 2.

The mean age of patients at the time of diagnosis of ApHCM was 47 ± 14 years, with 35 of 58 patients (60.3%) being male. In 49 of 58 patients (84%), there was no known family history of HCM, and only 20.7% had a family history of SCD. Approximately two-thirds of the patients (34/58; 58.6%) were hypertensive, 12/58 (20.7%) had a BMI ≥ 30 kg/m^2^, and 22% were asymptomatic. The majority of patients (76%) were diagnosed with ApHCM either due to symptoms such as heart palpitations (27.6%), chest pain (17%), or shortness of breath (17%), or during incidental screening (24%). Four patients (6.9%) had a history of myocarditis; two of them experienced chest pain, while the other two presented with palpitations. A pathogenic variant was detected in only one of these patients, in the MYH7 gene. One patient initially presented with syncope, and another with a resuscitated cardiac arrest at diagnosis. This latter patient, a 59-year-old man with an apical wall thickness of 18 mm and fibrosis on the cardiac magnetic resonance imaging (CMR), with no history of SCD or HCM, did not have any pathogenic genetic variant detected.

Regarding the baseline echocardiographic findings, the mean apical left ventricular wall thickness was 18.7 ± 4 mm. In this study, there were 42 of 58 patients (72.4%) with the pure ApHCM phenotype, and the remainder had the mixed phenotype. Left ventricular outflow tract obstruction was uncommon; it was not present in any of the cases. The mean left atrium size was 46.3 ± 5.5 mm, and the mean ejection fraction was 62 ± 7%. Apical aneurysm was observed in 17.2% (10/58) of cases. Patients with an apical aneurysm did not differ in complication rates compared to those without (5/10 vs. 16/48, *p* = 0.47) nor in the incidence of atrial fibrillation (5/10 vs. 19/48, *p* = 0.72). Genetic testing was performed in seven of these ten patients and in only one female patient with mixed type of apical hypertrophy, a pathogenic variant in MYH7, was detected Cardiac magnetic resonance imaging was performed in 46 patients (79.3%) of the cohort, and interestingly, 82.6% of them showed late gadolinium enhancement (LGE) uptake. Of those with LGE, 31.6% experienced ventricular arrhythmias on the monitor. All patients with an apical aneurysm who underwent CMR (8/10) exhibited signs of fibrosis. There was only one female patient with an apical wall thickness of 13 mm, “relative” ApHCM, who exhibited characteristic precordial T-wave inversion and a loss of the usual tapering of apical wall thickness on the CMR. No signs of fibrosis were observed, but the 24 h monitor revealed short episodes of non-sustained ventricular tachycardia, which were treated with β-blockers.

### 3.2. Events, Complications and Interventions of the HCM Cohort

The mean follow-up for this cohort was 17.2 years (±10.9, range 0–52 years). Overall, 21 of 58 patients (36.2%) had HCM-related events (Table 3) during their disease course, 6 of 58 patients (10.3%) had a stroke, 7 of 58 patients (12.1%) had heart failure and nearly 7% of the cases had a myocardial infarction. Atrial fibrillation was present in 24 of 58 patients (41.4%) and whereas non-sustained ventricular tachycardia occurred in 17 patients (29.3%). Most of the patients (92.3%) with ventricular arrhythmias depicted signs of fibrosis on the CMR. An ICD was placed in 13 of 58 patients (22.4%), and two of them had an appropriate ICD discharge. The female patient with a wall thickness of 16 mm experienced an ICD discharge while running due to sustained VT, and a pathogenic variant in the non-sarcomeric ALPK3 gene was detected. The other patient, a male with a mixed apical phenotype and a wall thickness of 28 mm, had no pathogenic genetic variant detected. Both patients showed signs of fibrosis on the CMR. β-blockade therapy as monotherapy was being administrated in 86.2% patients. In this study, only 15.5% had a family history of HCM, and 20.7% had a family history of SCD.

Four patients died during the follow-up period. One patient, an 81-year-old woman with an apical wall thickness of 22 mm, died of SCD, but she had no prior history of HCM or SCD, and no genetic testing was performed. Another woman, aged 88, died from a myocardial infarction; her apical wall thickness was 16 mm, and no genetic variant was detected. A 74-year-old man died from advanced heart failure; he had an apical wall thickness of 23 mm (mixed type) and an ICD due to previous unexplained syncope, but had no family history of HCM and no genetic test was performed. Lastly, a 64-year-old man died from frontotemporal dementia; he had an ICD, an apical wall thickness of 25 mm (mixed type), and a history of SCD, but no genetic variant was found. There was no significant difference in the prevalence of pure apical hypertrophy between males and females (25/35 vs. 17/23, *p* = 0.54). Similarly, no significant sex-based difference was observed in the prevalence of ventricular arrhythmias (males: 9/35 vs. females: 8/23, *p* = 0.3). Sex was not associated with a higher rate of HCM-related events (males: 14/35 vs. females: 7/23, *p* = 0.6). Additionally, there were no notable differences between males and females in terms of hypertension, diabetes, or the frequency of undergoing genetic testing. Overall, male and female patients showed comparable characteristics and outcomes.

### 3.3. Genetic Testing and Results of the HCM Cohort

From the 58 ApHCM index patients recruited to the study, 50 (86.2%) had genetic testing (Table 4 and Table 5). The main reported cause for not having genetic testing was the absence of a request by the cardiologist (13.8%). There was not a significant difference between the frequency of tested males (30 in 35, 86%) compared to females (20 in 23, 87%). Among the 50 patients tested, 7 (14%) were genotype positive (G+) with a pathogenic (P) or likely pathogenic (LP) variant (Table 6), primarily in sarcomeric genes: MYH7 (*n* = 3, 43%), MYBPC3 (*n* = 1, 14.2%), ALPK3 (*n* = 2, 28.6%), and MYL2 (*n* = 1, 14.2%).

In 13 patients, variants of unknown significance (VUS) were identified in the original analysis, but after re-analysis in 2025, only 4 variants remained classified as reportable VUSs, while the other 9 were reclassified as weak/unreportable VUS or benign. Among the four reclassified VUS, all had specific symptoms and findings on ECG or imaging. Two of the patients with a genetic result of VUS (in FLNC, MYBPC3) were found to have myocardial fibrosis on CMR without any explanation other than ApHCM. Another patient with a VUS was found to have apical aneurysm at 44 years of age. However, no genetic variant was detected in 39 out of 50 patients (78%). In a patient, a VUS was identified in the cardiac troponin I (TNNI3) gene, and a variant meeting the AMG criteria for classification as likely pathogenic was also detected in the skeletal muscle α-actin (ACTA1) gene, which is primarily associated with skeletal myopathy. Cardiac involvement in ACTA1-related myopathy is rare and has been reported in only a few cases, typically presenting as dilated cardiomyopathy [23,24]. This patient, though, showed no signs of myopathy and had an apical wall thickness of 17 mm, making it uncertain whether the ACTA1 variant contributes to the cardiac phenotype.

In most cases where a pathogenic (P) or likely pathogenic (LP) variant was detected, the patients were female, diagnosed at a young age, had a family history of either HCM or SCD, and exhibited signs of fibrosis on the cardiovascular magnetic resonance (CMR) imaging (Table 6). None of the identified variants were de novo, although parental testing for the variant in the ACTA1 gene has not yet been performed.

## 4. Discussion

The present study provides results regarding the frequency and distribution of disease-causing variants in a cohort of 58 unrelated patients with apical HCM who underwent genetic testing for clinical purposes at a tertiary referral center in Linköping in Sweden. Through comprehensive long-term clinical follow-up (a mean duration of approximately 17 years) and a re-evaluation of genetic data using AI-assisted tools, this study offers valuable insights into the heterogeneity and complexity of apical hypertrophic cardiomyopathy. In this population of ApHCM, 14% of patients were found to have disease-causing variants that predominantly occurred in genes encoding proteins for the thick sarcomere filament, with three variants affecting the MYH7 gene and one affecting the MYBPC3 gene. This study cohort consists of patients from the Southeast region of Sweden, with Linköping serving as a major tertiary referral center for genetic testing of hereditary cardiomyopathies. Similar results may be observed in other regions of Sweden and the four other tertiary centers. However, the accessibility of genetic testing may vary across different regions in Sweden and Europe, potentially introducing a referral bias. This bias could lead to higher rates of genetic testing in patients with a positive family history of HCM, thereby increasing the likelihood of identifying known disease-causing sarcomere genetic variants. The diagnostic yield of genetic screening for the identification of pathogenic variants in patients with HCM varies across studies, ranging from 30 to 63% [25,26]. The low percentage (14%) of patients with a positive genotype and apical hypertrophy in this study is somewhat puzzling, suggesting the involvement of other affected gene loci beyond the known sarcomere protein-encoding regions, especially in the case of a family history of SCD or HCM. Nevertheless, genetic testing remains a valuable tool in clinical practice for patients with apical HCM, as it may aid in confirming the diagnosis, especially in borderline cases.

To date, the literature reporting sarcomere protein gene variants in patients with apical HCM is limited to a relatively small number of unrelated patients. Genetic analyses of small-sized populations with ApHCM have identified a predilection for ACTC1 (cardiac α-actin) variants, indicating a direct causality of ApHCM with cardiac actin variants such as p.Glu101Lys and even with myosin essential light-chain pathogenic variants [16,20]. Olson et al. [19] and Mogensen et al. [27] suggest that variants on the myosin-exposed surface of cardiac actin may disrupt important electrostatic interactions, impair force generation, and trigger a hypertrophic response. In this cohort, a female patient with mixed apical hypertrophy (25 mm) and fibrosis on the CMR was found to have a pathogenic variant in the myosin light-chain-2 gene, MYL2, along with two VUSs in the MYH7 gene, which may contribute to the apical phenotype. Epstein et al. [28] suggest an alternative model in which mutated residues within the essential myosin light chain distort the stretch-activated response, an intrinsic property of muscle that leads to oscillatory power.

In a larger cohort of 71 patients with the apical form of HCM [18], the yield of genetic testing was relatively low, with only 25% of cases showing a positive result. The variants most commonly identified were in MYBPC3 and MYH7, similar to findings in the general HCM cohort [18]. In another study by Gruner et al. [17], 13% of patients (8/61) were found to have disease-causing sarcomere gene variants, predominantly in genes encoding proteins for the thick sarcomere filament. Five variants affected the MYH7 gene and two affected the MYBPC3 gene, similar to the findings of the present cohort. In contrast, in the study by Arad et al. [16], comprehensive genetic analyses of 15 probands with apical HCM identified six disease-causing variants (40%) in genes encoding β-myosin heavy chain, troponin I, cardiac actin, and myosin essential light chain. This provides molecular validation that hypertrophy localized to the cardiac apex is within the spectrum of morphologies triggered by sarcomere protein gene defects. Despite a shared molecular etiology, there are notable differences in the genetic and clinical profiles associated with apical and prototypic morphologies of HCM. First, patients with ApHCM are less likely to report a positive family history; in the current study, 15.5% had a family history of HCM, suggesting differences in pathophysiology. Additionally, in prior studies [25,26], a substantial fraction of HCM were associated with myosin-binding protein C variants; in this study, only one patient with ApHCM had a pathogenic variant in MYBPC3 gene. In contrast, myosin heavy-chain variants (MYH7) were the most commonly found (43%) in this cohort of patients with ApHCM. All three patients with pathogenic MYH7 variants were female, two of them had a mixed-apical phenotype with fibrosis on the CMR and only one had atrial fibrillation (Table 6). Thus, the distribution of sarcomere protein gene variants in this ApHCM cohort most frequently revealed mutations in the MYH7 gene, consistent with their well-established role in classical forms of HCM.

Furthermore, in this cohort, two patients had deleterious variants in a non-sarcomere-related gene, the ALPK3 gene coding for α-protein kinase 3 (ALPK3), which is a 1705 amino-acid-long atypical protein kinase predicted to play an essential role as a transcription factor during cardiomyocyte differentiation [29]. α kinase 3 (ALPK3)-associated hypertrophy primarily presents in the apical region, with ALPK3 heterozygous truncating variant (ALPK3tv) carriers showing a higher prevalence of apical and concentric patterns of hypertrophy [30,31,32]. In this study, a male patient with a pathogenic ALPK3 variant was diagnosed at the age of 25, showing pure apical hypertrophy with a wall thickness of 18 mm. He presented with palpitations, though no severe arrhythmias were detected. The other patient was an 18-year-old female with a mixed apical phenotype, 16 mm wall thickness, and fibrosis on the CMR. She presented with syncope and arrhythmias, for which she received an ICD. A recent study [32] suggests that heterozygous ALPK3-related HCM may result in a milder degree of hypertrophy compared to other Mendelian causes of HCM. However, the increased occurrence of apical aneurysms may have implications for ventricular arrhythmia risk. In that study [32], 21 individuals with ALPK3 heterozygous truncating variants were reported to have late-onset hypertrophic cardiomyopathy, often with frequent apical involvement and the presence of apical aneurysms. In contrast, in this cohort, patients with ALPK3 pathogenic variants—one with a nonsense coding variant where the reading frame is interrupted by a premature stop codon, and the other with a frameshift variant—presented with severe symptoms, primarily arrhythmias and typical features of ApHCM at a relatively young age, despite the presence of mild apical hypertrophy. This could be explained by the fact that the resulting protein product is much shorter and subject to nonsense-mediated decay (NMD), a cellular mechanism that prevents the expression of truncated proteins, leading to a loss of function of that protein and the earlier onset of disease in these patients. Our results suggest that the inclusion of the ALPK3 gene in genetic testing panels for all the types of HCM is essential.

A re-evaluation of the original raw data from genetic testing using AI-based software identified all previously reported likely pathogenic/pathogenic variants in the cohort as well as a previously unreported variant in ACTA1. The ACTA1 gene encodes the α-actin isoform, a sarcomeric protein essential for muscle contraction, particularly abundant in skeletal muscles [33]. Variants in ACTA1 associated with combined cardiac and skeletal myopathies have been reported, but ACTA1 represents only ~20% of the total actin pool in cardiomyocytes, making its role in cardiomyopathy controversial [33]. Although cardiac involvement is rare, some reported cases associate ACTA1 variants with cardiac conditions such as dilated cardiomyopathy (DCM) [23,24] and hypertrophy [34,35], often occurring jointly with myopathy. As one of six highly conserved actin isoforms, ACTA1 polymerizes to form filaments that interact with nebulin, troponins, and tropomyosins, establishing the core of the thin filament. Genetic variants that impair this contractile apparatus are associated with skeletal myopathies and cardiomyopathies. The majority of ACTA1 variants (approximately 74%) [36] are associated with nemaline myopathy; however, an increasing number have been linked to cardiomyopathy and emerging phenotypes such as distal myopathy. Of note, there has been a report of a variant in ACTA1 [37] that disrupts molecular contractility in cardiomyocytes through a mechanism involving troponin and tropomyosin, which is interesting considering that the patient in the current study also had a variant in the troponin gene TNNI3. Even small amounts of mutant ACTA1 protein incorporated into thin filaments can impair molecular contractility, and this disruption relies on the presence of troponin and tropomyosin. Thus, in this cohort, a female patient, without any skeletal myopathy but exhibiting pure apical hypertrophy and a likely pathogenic variant in ACTA1, as well as a VUS in TNNI3, suggests that despite α-actin being one of the most expressed proteins in skeletal muscle, it might produce myocardial involvement alone. Further studies are needed to confirm this. Our results highlight that re-evaluating genetic data using advanced AI-assisted tools may aid in identifying additional genetic variants that contribute to the ApHCM phenotype.

Still, genetic testing failed to define the cause of apical HCM in 39 probands even after re-evaluation. This may reflect that a single gene defect may not be the cause of apical HCM in some patients. The negative family history of HCM in 34 of the 39 patients, in whom no sarcomere variant was found, supports the idea that complex genetics and/or gene–environment interactions may contribute to this phenotype in certain individuals. However, a family history of HCM was present in 4 out of 7 genotype-positive patients with apical HCM. Genome-wide association studies could help identify additional causative or modifying genetic loci. Furthermore, ApHCM is characterized by an unusual pattern of hypertrophy, and the reasons behind this distinct distribution, as opposed to the more typical septal involvement, remain unclear. In a recent study [38], Yuan et al. evaluated the genetic underpinnings and clinical impact of focal hypertrophy; seventeen genome-wide associations for left ventricular mass (LVM) were identified, three unique associations with increased apical mass, and three additional unique associations with increased septal mass. A polygenic risk score (PRS) for apical and septal mass reaffirmed an elevated risk of atrial fibrillation, cardiomyopathy, and ICD implantation [38]. Subjects at the highest level of apical mass PRS had the greatest risk for cardiomyopathy compared with those at the highest level of total LVM and septal mass PRS. Distinguishing between subtypes of LV hypertrophy (LVH) may improve risk stratification and motivate targeted therapies.

The current guidelines [9,10,11] on HCM make a brief reference to ApHCM, without providing ApHCM-specific recommendations to guide diagnosis and management. Two phenotypes of ApHCM should be recognized and distinguished clinically because as studies [14,15] have highlighted they have different prognoses and genetic features. In this cohort, 42 patients had the pure apical form, while 16 had the distal-dominant form. Patients with the pure apical type experienced fewer adverse events related to cardiomyopathy compared to those with the mixed type (11/42 vs. 10/16, *p* = 0.015), and fewer had implanted ICDs (2/42 vs. 11/16, *p* < 0.001) but were more hypertensive (31/42 vs. 3/16, *p* < 0.001). Notably, those with distal-dominant hypertrophy developed more heart failure (7/16 vs. 0/42, *p* < 0.001), had more episodes of non-sustained ventricular arrhythmias on the monitor (11/16 vs. 6/42, *p* < 0.001) and were younger at diagnosis (40.2 ± 15.2 vs. 49.5 ± 12.6, *p* = 0.02). However, there was no significant difference in the detection rate of pathogenic or likely pathogenic variants in sarcomeric protein-encoding genes between the two groups (*p* = 0.09). This may be explained by the low percentage of patients with a positive genotype in both groups. These findings align with the understanding that mixed-type hypertrophy generally follows the course of classical HCM, while pure apical hypertrophy represents a more distinct phenotype.

In contrast to the other forms of HCM, ApHCM is considered as a ‘benign’ form of HCM, rarely associated with the occurrence of cardiovascular mortality and morbidity [5,7]. However, more recent studies [3,39] suggest that cardiovascular complications, such as myocardial infarctions and arrhythmias, are not uncommon even in ApHCM. Patients with ApHCM face higher rates of atrial fibrillation and apical aneurysms. In this cohort, only four patients died at relatively older ages, and two of them had a mixed type of apical hypertrophy. One of the deceased patients had an ICD for primary prevention due to ventricular arrhythmias; however, the cause of death was end-stage heart failure. In this study, atrial fibrillation (AF) was observed in 24 of 58 patients (41.4%), and 6 out of the 58 ApHCM patients (10.3%) experienced a stroke. Of these, three had AF, and one patient had a superior mesenteric artery occlusion, probably caused by AF. Of the 24 patients with AF, 7 (29%) had AF as the initial manifestation, which led to their diagnosis. The prevalence and annual incidence of AF in a ApHCM study by Joung [40] were reported to be 25.2% and 4.73% per year, respectively, which are comparable to a previous study by Olivotto et al. [41], where the prevalence of AF in HCM was 22%, with an annual incidence of 2%. However, their study included all types of HCM without specifying the proportion of each type. Eriksson et al. [5] reported a 12% prevalence of AF over a mean follow-up of 13.6 years in patients with pure ApHCM. No significant difference in AF prevalence was found based on sex (*p* = 0.28) or the type of apical hypertrophy (18/42 pure vs. 6/16 mixed, *p* = 0.8). Patients with AF had significantly larger left atrial (LA) dimensions (50 ± 4.5 mm vs. 43.7 ± 4.4 mm, *p* < 0.001) and were older (52.6 ± 10.4 years vs. 42.9 ± 14.8 years, *p* < 0.008). Previous studies [42], including those with all types of HCM, showed that an enlarged LA is not only a consequence of impaired diastolic function but also the strongest predictor of AF. Therefore, patients with ApHCM should undergo longitudinal follow-up with careful monitoring for AF development, and a more aggressive therapeutic approach should be considered when AF occurs.

Patients with a positive genotype were more likely to have a positive family history for HCM than were patients with a negative genotype (4/7 vs. 5/39, *p* = 0.020). Otherwise, there were no further differences present, especially no higher incidence of HCM-related events compared with the patients with a negative genotype (3/7 vs. 14/39, *p* = 0.5). The genotype was not associated with a higher rate of HCM-related events including stroke, atrial fibrillation, ventricular arrhythmias, heart failure, or ICD implantation. Furthermore, the percentage of patients with the distal-dominant form was not significantly higher in the genotype-positive group compared with the genotype-negative group. The lack of difference in terms of the apical morphology pattern (pure apical versus distal dominant) suggests that the left ventricular muscle mass may not be purely associated with genotype but rather with the interaction of other background modifier genes and/or hemodynamic factors.

In classical hypertrophic cardiomyopathy, several studies [43,44] have demonstrated that identifying pathogenic variants in sarcomere genes provides valuable prognostic information, with a higher risk of adverse HCM-related outcomes observed in patients carrying these variants. In contrast, findings from Towe et al. [18] suggest that, in patients with apical HCM, there is no significant difference in clinical outcomes between genotype-positive and genotype-negative individuals. However, it is important to note that their analysis included non-cardiac-related deaths among the adverse events, which may have confounded the relationship between genotype status and HCM-specific clinical outcomes. In our study of this ApHCM cohort, we also did not find a significant difference in the incidence of HCM-related events based on genotype status. This may be partly due to the relatively small number of patients with a positive genotype.

On the other hand, the study by Sugiura et al. [15], which examined a comprehensive cohort of 110 patients with ApHCM, observed a significantly higher incidence of adverse events in the genotype-positive group compared to the genotype-negative group. These findings suggest that the presence of sarcomere gene variants could serve as a potential predictor of worse prognosis, even within the apical HCM phenotype, though no definitive prognostic stratification has yet been established. While the presence of pathogenic variants has been associated with worse outcomes in classical HCM, recent data indicate that individual genetic variants do not reliably predict prognosis in the majority of HCM patients [43]. Therefore, clinical decisions and a patient’s prognosis should not be solely based on the presence of specific sarcomere variants, regardless of the type of hypertrophy.

In the future, polygenic risk scoring or the identification of specific variants linked to arrhythmia burden may improve risk stratification. However, at present, clinical and imaging features remain superior predictors of prognosis in ApHCM. Notably, fewer ApHCM patients report a positive family history compared to those with classical HCM, which challenges the applicability of conventional HCM risk stratification models [9] that heavily rely on family history of SCD. Certain clinical and imaging features have been associated with increased cardiovascular morbidity in ApHCM and should be carefully considered during risk assessment. These include mixed ApHCM morphology, younger age at presentation, complete end-systolic cavity obliteration at the level of the papillary muscles, the presence of apical aneurysms, extensive late gadolinium enhancement (LGE) on cardiac magnetic resonance imaging, documented arrhythmias, and unexplained syncope [15,45]. Incorporating these factors into individualized clinical evaluation may improve prognostic accuracy and inform management strategies in this unique HCM subtype.

## 5. Conclusions

ApHCM, the least common subtype of HCM, was associated with a negative genetic test result in 78% of cases. Among those with a positive genetic test, the most common genotypes were found in the MYH7 gene, which was identified in 43% of patients and in the non-sarcomeric ALPK3 gene (28.6%). These findings suggest that other affected gene loci, beyond the known sarcomere protein-encoding regions, may be involved, and genome-wide association studies could help identify additional causative or modifying genetic factors.

## 6. Limitations

Inherent limitations of retrospective, observational studies. The low number of events limited the ability to assess the independent effect of genotype or compare the effects of the pathogenic genetic variants detected in these ApHCM patients. Consequently, the low number of events makes any multivariable analysis challenging.

We believe this paper serves as a guide to the genotype status of HCM patients with the apical subtype in Sweden and there is an imperative need of multi-center studies reporting phenotype and genotype of these subtype of hypertrophy for improvement of management and risk stratification. We are aware that the statistical power, with only seven patients having a positive genotype, is limited. However, this is the largest Swedish cohort of ApHCM with genetic testing that has been reported to date, and, importantly, ApHCM has a good prognosis compared with the other morphologies, which implies a lower event rate.

## Figures and Tables

**Table 1:** Baseline characteristics and reasons for the initial evaluation of the study participants.

**Baseline characteristics**	
Age at diagnosis, mean (SD, min–max)	46.91 ± 13.9 (18–70)
Sex, male	35/58 (60.3%)
Hypertension	34/58 (58.6%)
Angina	9/58 (15.5%)
Diabetes	9/58 (15.5%)
Smoking	14/58 (24.1%)
BMI kg/m^2^	26.8 ± 4 (20–39)
**Reason for diagnosis**	
Symptoms	44/58 (75.9%)
Check-up/Incidental	14/58 (24.1%)
History of pericarditis/myocarditis	4/58 (6.9%)
**Presentation**	
Family history of HCM	9/58 (15.5%)
Family history of SCD	12/58 (20.7%)
**Symptoms at initial evaluation**	
Dyspnea	10 (17.2%)
Chest pain	10 (17.2%)
Palpitations	16 (27.6%)
NYHA class III/IV	2 (3.4%)
Syncope	1 (1.7%)
Resuscitated cardiac arrest	1 (1.7%)
Tiredness	5 (8.6%)
No symptoms at all	13 (22.4%)
Abnormal SBP response to exercise	5 (8.6%)
Murmur	11/58 (19%)

Abbreviations: SD, standard deviation; BMI, body mass index; HCM, hypertrophic cardiomyopathy; SCD, sudden cardiac death; NYHA, New York Heart Association of Stages and Classification of Heart Failure.

**Table 2:** Baseline characteristics of the initial echocardiographic evaluation of the study participants.

**Follow-up**	
Atrial fibrillation	24/58 (41.4%)
NSVT	17/58 (29.3%)
Syncope	7/58 (12.1%)
**Interventions**	
ICD	13/58 (22.4%)
Complications	21/58 (36.2%)
Stroke	6/58 (10.3%)
Myocardial infarction	4/58 (6.9%)
Heart failure	7/58 (12.1%)
Arterial embolism	1/58 (1.7%)
Appropriate ICD discharges	2/13 (15.4%)
Resuscitated cardiac arrest	1/58 (1.7%)
SCD	1/58 (1.7%)
**Mortality**	
Overall mortality	4/58 (6.9%)
Cardiovascular mortality	3/58 (5.2%)
**Medications**	
Betablockers	50 (86.2%)
Verapamil	3 (5.2%)

Abbreviations: SD, standard deviation; LVEF, left ventricular ejection fraction; LVOT, left ventricular outflow tract; LA, left atrium; CMR, cardiac magnetic resonance imaging.

**Table 3:** Follow-up data, events, interventions, complications and mortality of the hypertrophic cardiomyopathy (HCM) cohort.

**Follow-up**	
Atrial fibrillation	24/58 (41.4%)
NSVT	17/58 (29.3%)
Syncope	7/58 (12.1%)
**Interventions**	
ICD	13/58 (22.4%)
Complications	21/58 (36.2%)
Stroke	6/58 (10.3%)
Myocardial infarction	4/58 (6.9%)
Heart failure	7/58 (12.1%)
Arterial embolism	1/58 (1.7%)
Appropriate ICD discharges	2/13 (15.4%)
Resuscitated cardiac arrest	1/58 (1.7%)
SCD	1/58(1.7%)
**Mortality**	
Overall mortality	4/58 (6.9%)
Cardiovascular mortality	3/58 (5.2%)
**Medications**	
Betablockers	50 (86.2%)
Verapamil	3 (5.2%)

Abbreviations: NSVT, non-sustained ventricular arrhythmia; ICD, implantable cardioverter defibrillator; SCD, sudden cardiac death.

**Table 4:** Genetic testing and results of the apical hypertrophic cardiomyopathy (ApHCM) cohort.

**Genetic Testing**	50/58 (86.2%)
**Result of Genetic test**	
Pathogenic/Likely pathogenic	7/50 (14%)
No genetic variant found	39/50 (78%)
Variants of unknown significance (VUS)	4/50 (8%)
**Genetic Variants (Pathogenic/Likely Pathogenic)**	
MYBPC3	1/7 (14.2%)
MYH7	3/7 (43%)
ALPK3	2/7 (28.6%)
MYL2	1/7(14.2%)

G+: genotype positive, G−: genotype negative. Comprehensive genetic analyses of 50 probands with apical HCM identified 7 disease-causing variants in genes encoding the β (β)-myosin heavy chain (MYH7), cardiac myosin-binding protein C (MYBPC3), Myosin regulatory light chain 2 (MYL2), and α-protein kinase 3 (ALPK3). Thus, only in 14% of cases, and mostly sarcomeric gene defects specifically produced this distinctive HCM morphology.

**Table 5:** The genetic variants observed in the apical HCM patients recruited from the Southeast region of Sweden between 2011 and 2024 were assessed according to the American College of Medical Genetics and Genomics (ACMG) guidelines. All of these variants were detected in a heterozygous state. In two unrelated cases, the same likely pathogenic (LP) variant was found in the MYH7 gene. In one case, in addition to the LP variant, variants of unknown significance (VUS) were also identified, though it remains unclear whether they contribute to the cardiac phenotype. Additionally, a LP variant in the α-skeletal isoform of actin (ACTA1) was detected, but the patient did not exhibit any signs of functional skeletal myopathy, making it uncertain whether this variant is linked to the apical HCM phenotype. “Hot” VUSs are also listed in the table—these are variants that narrowly missed a “likely pathogenic” classification and require further investigation to gather additional evidence for a more definitive classification. The Emedgene software identified the four VUSs mentioned above. The other nine variants previously classified as VUS are not reported here due to the implementation of stricter classification criteria.

**Genetic Variants (Pathogenic, P/Likely Pathogenic, LP)**
NM_000256.3(MYBPC3):c.3043dup (LP) NM_000257.3(MYH7)c.5135G > A (P) NM_000257.4(MYH7):c.427C > T (LP) NM_000257.4(MYH7):c.427C > T (LP)
**NM_000432.3(MYL2):c.173G > A (P)** + NM_000257.3(MYH7):c.4031G > A(VUS) + NM_000257.3(MYH7):c.4481A > T (VUS)
NM_020778.5(ALPK3):c.676C > T (LP) NM_020778.5(ALPK3):c.3726del p.(Lys1243Argfs*29)(P)
**Genetic variants (Uncertain significance, ‘Hot’ VUS)** ▪ NM_000363.5(TNNI3):c.484C > T(VUS) + NM_001100.4(ACTA1):c.400del (p.Met134fs)(LP) ▪ NM_000258.3(MYL3):c.383G > A ▪ NM_001458.5(FLNC):c.5727_5729del ▪ NM_000256.3(MYBPC3):c.2441_2443del

**Table 6:** Specific genetic variants and clinical and echocardiographic features of patients with a positive genotype. In this cohort, nine patients had pathogenic or likely pathogenic variants. In two unrelated cases, the same variant in MYH7 gene was detected. One patient with a pathogenic variant in the MYL2 gene also had two other VUSs in MYH7. In four patients, VUSs were identified, where in one case a variant in TNNI3 was detected as well as a likely pathogenic in skeletal ACTA1.

Genetic Variant P/LP	Age at Diagnosis/ Ethnicity	Sex	Presenting Symptoms	Family History of HCM	Family History of SCD	MWT (mm)	PURE APICAL Type	ICD	Atrial Fibrillation	Apical Aneurysm	Fibrosis CMR
**MYH7** NM_000257.3: c.5135G > A (P)	24, Syrian	F	Dyspnea	yes	yes	21	no—mixed	yes	no	yes	yes
**MYH7** NM_000257.4: c.427C > T (LP)	27, Sweden	F	Palpitations	yes	yes	18	yes	no	yes	no	no
**MYH7** NM_000257.4: c.427C > T (LP)	53, Sweden	F	ECG changes	no	no	17	no—mixed	no	no	no	yes
**MYBPC3** NM_000256.3: c.3043dup (LP)	53, Iran	M	NYHA 3	yes	no	24	yes	no	no	no	yes
**MYL2** NM_000432.3: c.173G > A (P) + MYH7 NM_000257.3: c.4031G > A(VUS) + MYH7 NM_000257.3: c.4481A > T (VUS)	35, Croatia	F	ECG changes	no	yes	25	no—mixed	no	no	no	yes
**ALPK3** NM_020778.5: c.676C > T p.(Arg226*)(LP)	25, Sweden	M	Palpitations	yes	no	18	yes	no	no	no	no
**ALPK3** NM_020778.5: c.3726del p.(Lys1243Argfs*29)(P)	18, Sweden	F	Syncope	no	no	16	no—mixed	yes	no	no	yes
**VUS**											
**TNNI3** NM_000363.5: c.484C > T(VUS) +ACTA1 NM_001100.4: c.400del(p.Met134fs)(LP)	47, Sweden	F	Dyspnea, no signs of skeletal myopathy	no	yes	17	yes	no	no	no	no
**MYL3** NM_000258.3: c.383G > A	44, Sweden	M	ECG changes	no	no	15	yes	no	no	yes	Not performed
**FLNC** NM_001458.5: c.5727_5729del	67, Sweden	M	Palpitations	no	yes	16	yes	no	yes	no	yes
**MYBPC3** NM_000256.3: c.2441_2443del	19, Sweden	M	Palpitations	no	no	20	no—mixed	yes	no	no	yes

Abbreviations: MWT, maximal wall thickness; HCM, hypertrophic cardiomyopathy; SCD, sudden cardiac death, ICD, implantable cardioverter defibrillator; F, female; M, male; NYHA, New York Heart Association Classification; P, pathogenic; LP, likely pathogenic; VUS, variant of uncertain (or unknown) significance; MYH7, β-myosin heavy chain; MYBPC3, myosin-binding protein C; MYL2, myosin light chain 2; ALPK3, α-protein kinase 3; TNNI3, cardiac troponin I; ACTA1, actin α 1;MYL3, myosin light chain 3; FLNC, Filamin-C.

## Data Availability

The original contributions presented in this study are included in the article. Further inquiries can be directed to the corresponding author. Informed consent was obtained from all subjects involved in the study. Written informed consent has been obtained from the patient(s) to publish this paper.
